# STA regulates succinylated AflM triggered by SCS to contribute to aflatoxin biosynthesis through the Ach1

**DOI:** 10.1080/21505594.2025.2532812

**Published:** 2025-07-18

**Authors:** Rui Xie, Zhenhong Zhuang, Qionghui Chen, Chunlan Xie, John Adejor, Xinyi Nie, Shihua Wang

**Affiliations:** State Key Laboratory of Ecological Pest Control for Fujian and Taiwan Crops, Fujian Key Laboratory of Pathogenic Fungi and Mycotoxins, and School of Life Sciences, Fujian Agriculture and Forestry University, Fuzhou, China

**Keywords:** *Aspergillus flavus*, succinyl-CoA synthetase, succinylation level, aflatoxin B1, development

## Abstract

*Aspergillus flavus* and its secondary metabolites, aflatoxins (AFs), especially aflatoxin B1 (AFB1), seriously affect agricultural production, food storage, and human health. Succinyl-CoA synthase ADP-forming subunit β (SCS) is involved in the synthesis of succinate from succinyl-CoA in the tricarboxylic acid cycle. In this study, we demonstrated that SCS led to decreased aflatoxin production. Bioassay results showed that deletion of *sucB* (the gene coding for SCS) led to increased succinyl-CoA accumulation. Catalyzed by succinyl transferase (STA), the increased amount of succinyl-CoA in Δ*sucB* leads to increased levels of global protein succinylation, which causes upregulation of AFB1 accumulation in Δ*sucB*. To elucidate the mechanism of increased AFB1 accumulation in Δ*sucB*, the relevant enzymes and metabolites involved in the aflatoxin biosynthesis pathway were examined through proteome and metabolome analyses. These data illustrate that the deletion of *sucB* results in an increase in (1’S, 5’S) – averufin catalyzed by AflK, (1’S)-averantin catalyzed by AflD, and aflatoxin G2/O- methylsterigmatocystin catalyzed by AflP. We also found that AflM is not only upregulated but also succinylated in Δ*sucB*; Ach1 (acetyl-CoA hydrolase, Ach1) is downregulated in Δ*sucB* and interacts with SCS. Therefore, we deduce a pathway of Ach1/STA-SCS-succinylated AflM for AFB1 biosynthesis, which provides knowledge for the control of *A. flavus* and AFs.

## Introduction

Two major mechanisms can expand the coding capacity of eukaryotic genomes, leading to the generation of diverse corresponding proteomes [1]. The first route is mRNA splicing at the transcriptional level, and the other is covalent posttranslational modification (PTM). PTM, an efficient biological regulator that links metabolism to protein and cell functions [2], has been a widespread concern for researchers. PTM occurs after RNA is translated into protein, which is mainly responsible for the modification of the side chains or backbones based on specific enzyme catalysis [1]. Lysine residues are modified by numerous groups of succinyl, methyl, and small ubiquitin-like modifiers (SUMO) *etc*. Not only can lysine residues with positively charged side chains covalently bind with succinyl groups, but they are also involved in various noncovalent interactions, including hydrogen bonds, electrostatic interactions, and van der Waals interactions [3]. Correspondingly, charge neutralization of the basic side chain of lysine residues inevitably triggers a battery of significant changes in protein function.

In the past several decades, some studies have worked on lysine succinylation modification, mainly focusing on the identification of succinyl-lysine sites. Weinert et al. utilized the new identification method of antibody affinity enrichment-strong cation exchange (SCX) chromatography-HPLC/MS/MS (HPLC: High Performance Liquid Chromatography; MS: mass spectrometry) and detected 2572 succinyllysine sites on 990 proteins in *E. coli* [4]. In addition, succinyllysine sites of proteins in *A. flavus* were identified by highly accurate nano-LC-MS/MS in combination with the enrichment of anti-succinyllysine antibody in our group, and 985 succinyllysine sites on 349 proteins were identified in *A. flavus* [5]. In *S. cerevisiae*, lysine residues are mutated to alanine (A) and arginine (R) to prevent succinylation, whereas glutamic acid (E) mimics succinylated lysine [6]. H4K31E can significantly reduce cell viability, whereas other mutations (H4K31A or H4K31R) do not affect cell viability in *S. cerevisiae* [6]. Another site, K77 on histone H4 was mutated to E, which can cause a loss of silencing at the telomere and rDNA [6]. In *A. flavus*, an aflatoxin biosynthesis-related protein, AflE, which had been identified as a succinylated protein by Ren et al., was mutated to study phenotypes [5]. AflEK370R or AflEK370A, which mimics the desuccinylation status of AflE, can decrease sclerotial production and aflatoxin B1 biosynthesis compared to the wild type (WT) in *A. flavus* [5]. Another protein, acetyl-CoA carboxylase (ACC, encoded by *accA*), was mutated to E to explore the effect of lysine succinylation of ACC on *A. flavus* phenotypes [7]. Collectively, the above studies demonstrated that succinylation indeed leads to different phenotypic consequences, which is necessary to deeply explore the succinylation phenomenon.

Succinyl-CoA is an intermediate metabolite involved in several important metabolic pathways, including the TCA cycle, catabolism of odd-chain fatty acids, and some branched-chain amino acids [8]. In *S. cerevisiae*, succinyl-CoA is formed by the regulation of α-ketoglutarate dehydrogenase complex (encoded by *kgd1*, *kgd2*, and *lpd1*) and succinyl-CoA synthetase/ligase (encoded by *lsc1* and *lsc2*), which is recognized as the succinyl donor to lysine that affects succinylation levels [4]. Kgd1 of α-ketoglutarate dehydrogenase complex and Lsc1 of succinyl-CoA ligase are important for the corresponding enzymatic activities, which are successfully induced in galactose-containing media [9,10]. Loss of *kgd1* can reduce global succinylation levels whereas loss of *lsc1* can increase global succinylation levels relative to WT in galactose-containing media [4]. Simultaneously, the changes of succinylation levels in the above statements depend on succinyl-CoA concentration [4,11]. In addition, *E. coli* can convert succinate to succinyl-CoA using succinyl-CoA synthetase to increase succinylation [8]. In human neurons and neuronal cell lines, E2k of KGDHC, which is responsible for the formation of dihydrolipoyl succinyltransferase of α-ketoglutarate dehydrogenase complex (KGDHC), functions as a succinyltransferase [12]. Besides, dihydrolipoamide succinyltransferase (DLST), an STA in *Yarrowia lipolytica* was deeply explored through the overexpressed or directed-site mutated methods [13]. The overexpression of DLST can disrupt the KGDH’s catalytic integrity which further affected α-ketoglutarate (α-KG) yields, and the directed-site mutation of Asp432 on *Yarrowia lipolytica’s* DLST to glutamate resulted in an increase of extracellular α-KG [13].

The influence of succinyl-CoA synthetase on metabolism was also reported by Lancaster *et al*.. A mouse model demonstrated that the deficiency of forebrain-specific succinyl-CoA synthetase (SCS) can significantly alter metabolites’ concentrations of methylmalonic acid (MMA), succinyl-CoA, amino acids (glutamate, taurine, aspartate, glutamine, and asparagine), and acyl-carnitines, indicating the interference of amino acid metabolism involved in TCA cycle and acyl-carnitines formation, while also mirroring patient’s biochemical phenotype caused by metabolic perturbations [14]. In *S. cerevisiae*, acetyl-CoA hydrolase (which catalyzes the hydrolysis of acetyl-CoA), termed Ach1p, is a mitochondrial enzyme that is indirectly involved in pseudohyphal differentiation [15]. In *Neurospora crassa*, *acu-8* was denoted as acetyl-CoA hydrolase [16,17], and the growth of the *acu-8* mutant, which is apparently deficient in acetyl-CoA hydrolase, was completely inhibited at high acetate concentrations (40 mM), whereas the growth inhibition was not significant at low acetate concentrations (4–16 mM) [16].

*Aspergillus flavus* (*A. flavus*), a saprophytic mold, is distributed worldwide. Not only can *A. flavus* cause human invasive aspergillosis, but also lead to chronic granulomatous sinusitis, keratitis, and cutaneous aspergillosis, which are extremely harmful to human health [18]. Additionally, *A. flavus* contaminates agricultural crops such as peanuts, cotton, and rice [19]. Aflatoxins (AFs), a class of *A. flavus* secondary metabolites (SM), are synthesized on a 70 kb gene cluster that encodes approximately 27 enzymatic reactions [20–23]. AflM is an important protein in AFB1 biosynthesis and is responsible for the conversion of versicolorin A to demethylsterigmatocystin [24–26]. Although the relationship between succinylation levels and succinyl-CoA generated by α-ketoglutarate dehydrogenase complex/succinyl-CoA ligase has been studied in *S. cerevisiae* and other species, related studies in *A. flavus* have not been conducted. In this study, we first explored the influence of SCS on *A. flavus* phenotypes and discovered related proteins (STA and Ach1) with SCS by multiple analysis methods. Ultimately, we clarified the biofunction and regulatory mechanism of SCS, which is involved in development and aflatoxin biosynthesis of *A. flavus*.

## Results

### Bioinformatics analysis of *A. flavus* SCS

**Figure 1.**
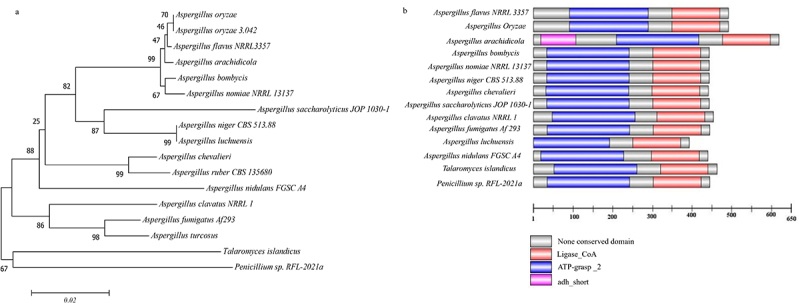
Identification of succinyl-CoA synthetase beta subunit (SCS) in *A. flavus*. (a) Phylogenetic trees of SCS in *A. flavus*. (b) domain analysis of SCS in different species.

*A*. *flavus* succinyl-CoA synthetase is composed of alpha and beta subunits. In this study, a putative succinyl-CoA synthetase beta subunit (SCS, accession number: G4B84_011127) was chosen because it can bind the substrate succinate and provide nucleotide specificity of the enzyme (succinyl-CoA synthetase). Phylogenetic analysis revealed that *A. flavus* SCS displayed high similarity to orthologs of *A. oryzae, A. oryzae* 3.042, *A. arachidicola*, *A. bombycis*, and *A. nomius* NRRL13137, and the *A. oryzae* SCS presented the highest similarity to *A. flavus* SCS (Figure 1a). The SCS domains in *A. flavus* and other fungal species are shown in Figure 1b, and all species contained conserved ATP_grasp and ligase-CoA domains. *A. arachidicola* contains another adh_short domain, except for the domains of ATP_grasp and ligase-CoA. The ATP_grasp domain contains conserved motifs of the phosphate binding loop and the Mg^2+^ binding site. The α- and β-subdomains of the ATP_grasp domain can form a fold that is responsible for grasping ATP. The ligase-CoA domain is found in α- and β-chains of succinyl-CoA synthetase, which can utilize ATP to form ADP. The above results indicated that SCS might be an important protein in *A. flavus* NRRL3357 and other *Aspergillus* genera (Figure 1a,b).

**Figure:**
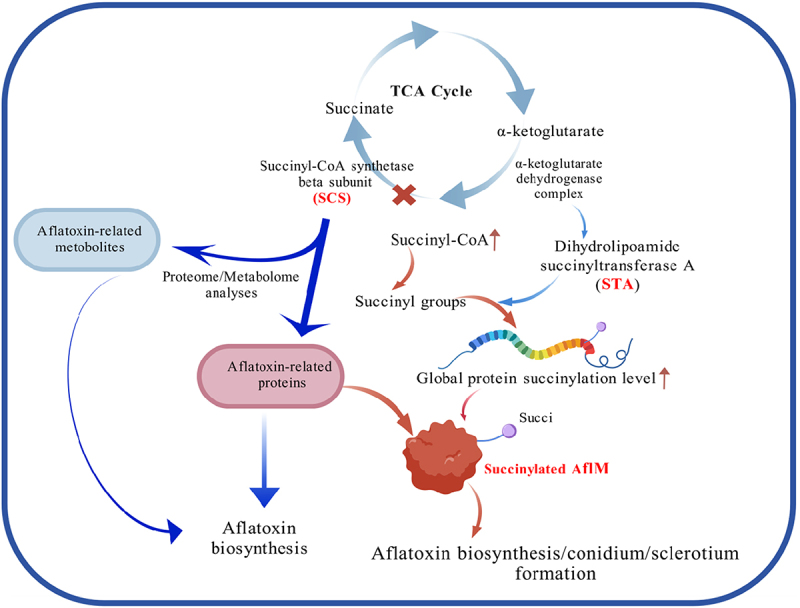

### Global succinylation level affected by SCS is associated with aflatoxin B1 biosynthesis

Before studying SCS function, gene knockout strain of succinyl-CoA synthetase subunit β (Δ*sucB*) and complementary strain (Δ*sucB*-com) were constructed and verified. According to Figure S1, the construction schematic of the deletion mutant and the principal diagram of the Southern blot are shown in Figures S1a,c. As shown in Figure S1b,d, the knockout strain Δ*sucB* was successfully constructed by PCR and Southern blot validation. Similarly, qRT-PCR results showed that the expression level of SCS in Δ*sucB* could not be detected compared to that of WT and Δ*sucB-*com (Figure S1E).

**Figure 2.**
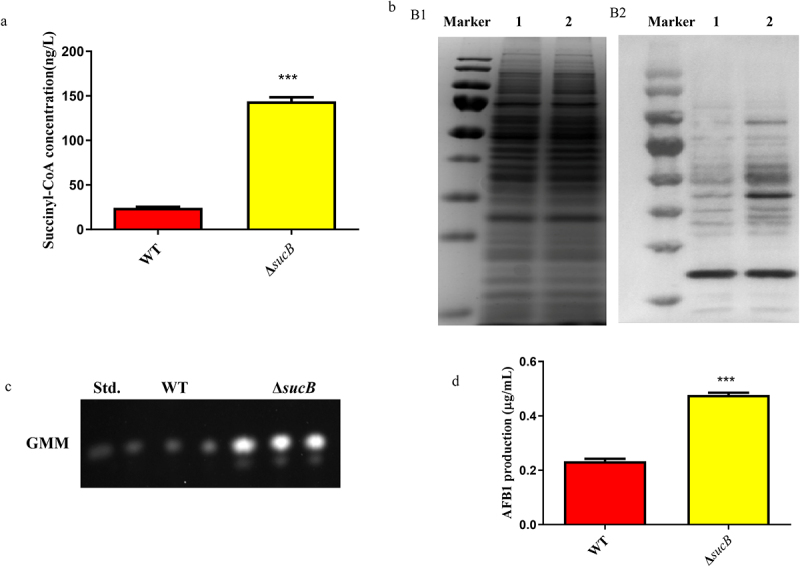
Total succinylation levels were affected by succinyl-CoA concentration. (a) succinyl-CoA concentrations of WT and Δ*sucB*. (b) global protein succinylation in WT and Δ*sucB* detected by western blot. (B1) Coomassie brilliant blue gel shows the same loading amounts. (B2) succinylation level of WT and Δ*sucB* (lane 1: WT, Lane 2: Δ*sucB*). (c) AFB1 production of WT and Δ*sucB* detected by TLC (d) AFB1 accumulation of WT and Δ*sucB* grown on GMM (***: *p* < 0.001).

In Figure 2a, deletion of *sucB* resulted in the upregulation of succinyl-CoA content. Correspondingly, the global succinylation level in Δ*sucB* was higher than that in WT (Figure 2b). Furthermore, AFB1 production in Δ*sucB* was measured, and the results shown in Figure 2c,d indicate that the deletion of *sucB* can cause more AFB1 accumulation compared to WT. Collectively, these results demonstrate that *sucB* gene deletion can regulate the global succinylation level of *A. flavus* proteins by affecting succinyl-CoA content, which finally causes changes in AFB1 production.

### SCS is critical for *A. flavus* development and aflatoxin B1 biosynthesis

**Figure 3.**
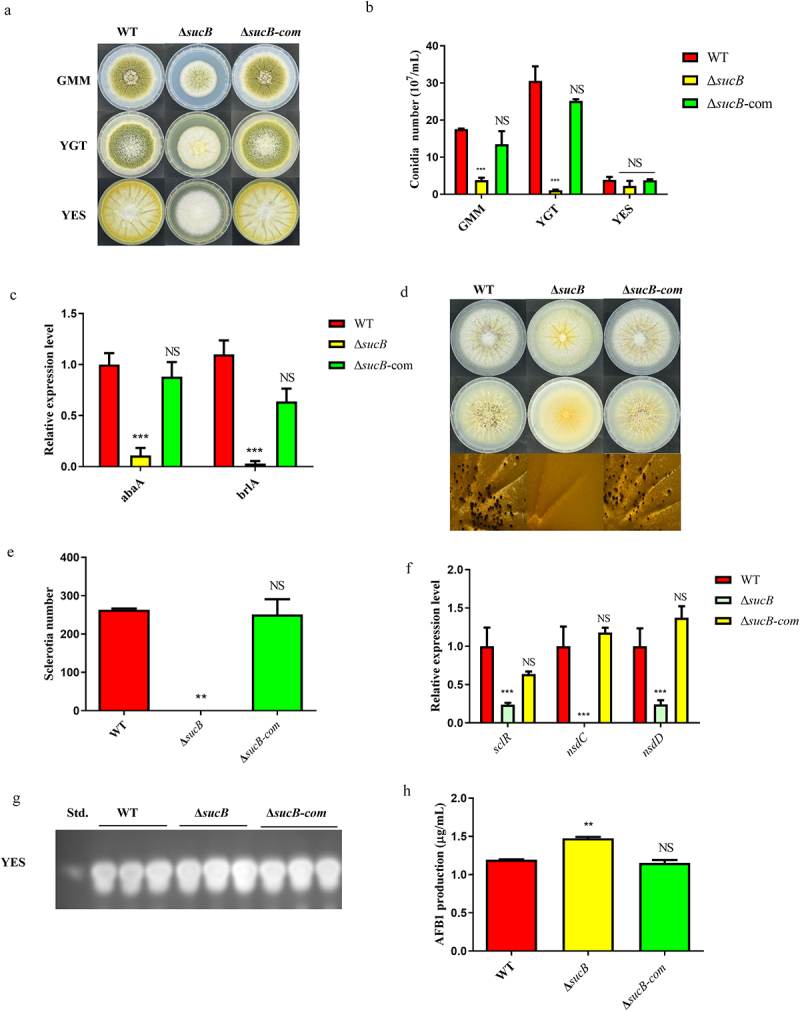
*SucB*’s effect on *A. flavus* phenotype. (a) vegetative growth of WT, Δ*sucB*, and Δ*sucB*-com strains on GMM, YGT, and YES media. (b) conidial amounts of WT, Δ*sucB*, and Δ*sucB*-com strains grown on different media. (c) relative expression levels of conidium-related genes *brlA* and *abaA* in various strains of *A. flavus*. (d) sclerotium formation of WT, Δ*sucB*, and Δ*sucB*-com in CM medium at 37°C for 7 days. (e) the number of sclerotia of WT, Δ*sucB*, and Δ*sucB*-com on CM medium. (f) relative expression level of sclerotium-related genes (*SclR*, *nsdC*, and *nsdD*) in WT, Δ*sucB*, and Δ*sucB*-com strains. (g) AFB1 production was measured by TLC after cultured in YES media at 29°C for 7 days. (H) quantification of aflatoxin production when WT, Δ*sucB*, and Δ*sucB*-com were cultured in YES media. (**: *p* < 0.01, and ***: *p* < 0.001, NS: not significant compared to WT).

SCS is an important enzyme associated with generating succinyl-CoA, so its bio-functions were further studied in this study. As shown in Figure 3a, the growth diameter of Δ*sucB* was smaller than that of the WT and Δ*sucB*-com on the GMM media. The spore amounts of Δ*sucB* on GMM, YGT and YES media were significantly decreased compared to that of WT and Δ*sucB*-com (Figure 3b). The relative expression levels of conidium-related genes *abaA* and *brlA* in Δ*sucB* were decreased compared to that of WT and Δ*sucB*-com (Figure 3c). Similarly, SCS also affected *A. flavus* sclerotial formation and AFs biosynthesis. The results revealed that the sclerotial production of Δ*sucB* was significantly downregulated relative to that of WT and Δ*sucB-*com (Figure 3d,e), and the expression levels of sclerotium-related genes *sclR*, *nsdC* and *nsdD* in Δ*sucB* were also significantly decreased compared to those in WT and Δ*sucB-*com (Figure 3f). The aflatoxin B1 (AFB1) accumulation was detected in this study, and the results indicated that AFB1 accumulation in Δ*sucB* was upregulated compared to that in WT and Δ*sucB-*com (Figure 3g,h). These data indicate that SCS affects conidial production, sclerotial formation, and AFB1 biosynthesis in *A. flavus*.

### STA is related to SCS and has the function of transferring succinyl group

**Figure 4.**
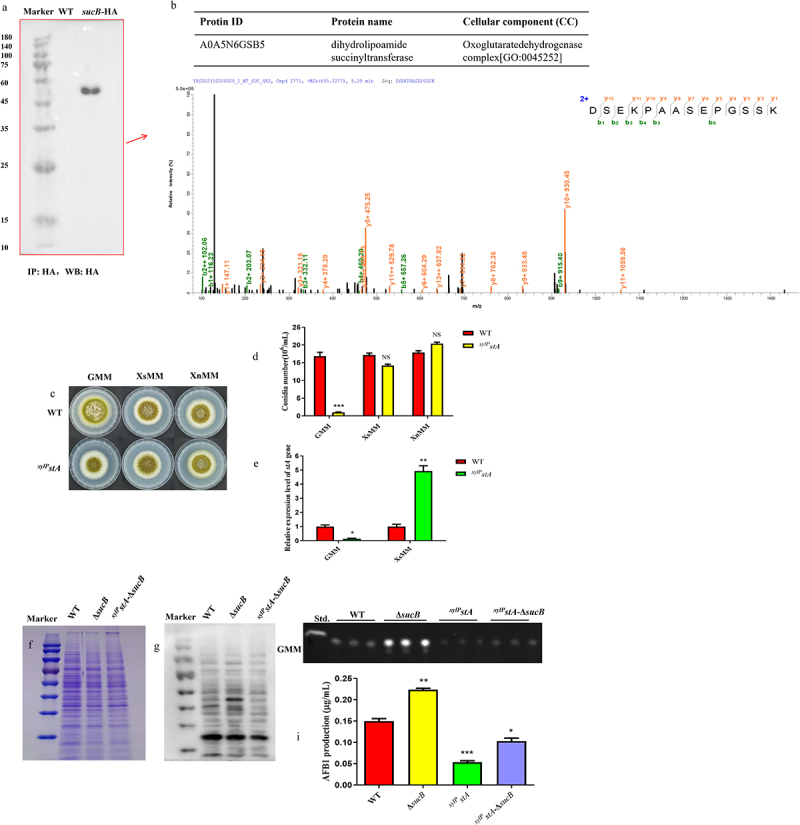
STA interacts with SCS and can transfer succinyl groups. (a) IP enrichment result of sucB-HA. (b) mass spectrum of dihydrolipoamide succinyltransferase that interacted with SCS in *A. flavus*. (c) colonies formed by WT and ^*xylP*^*stA* on GMM, XsMM, and XnMM agar plates. (d) conidia number of WT and ^*xylP*^*stA* strains grown on GMM, XsMM, and XnMM. (e) relative expression level of *stA* gene in WT and ^*xylP*^*stA* grown on GMM and XsMM agar plates. (f) total protein stained with Coomassie brilliant blue was as a control for protein loading volume. (g) measurement of succinylation level of total protein using the anti-succinyllysine mouse monoclonal antibody. (h) AFB1 production of WT, Δ*sucB*, and ^*xylP*^*stA*-Δ*sucB* was measured by TLC after cultured in GMM media at 29°C for 7 days. (i) AFB1 quantity of WT, Δ*sucB*, and ^*xylP*^*stA*-Δ*sucB* grown on GMM media (*: *p* < 0.05, **: *p* < 0.01, ***: *p* < 0.001, NS: not significant compared to WT).

Succinylated proteins exist in *A. flavus*, and succinyltransferase is indispensable for the generation of succinylated proteins. Dihydrolipoamide succinyltransferase A (STA; Accession number: G4B84_009564) is the E2 component of α-ketoglutarate dehydrogenase complex in TCA, which catalyzes α-ketoglutarate to generate succinyl-CoA. In this study, STA is explored for its putative succinyltransferase function. The SCS with three HA tags (*sucB*-HA) was constructed and successfully verified through PCR validation and DNA sequencing (Figure S2a,b). Based on the result in Figure 4a, the SCS protein was successfully enriched in the Co-IP and western blot experiments. Mass spectrometry (MS) revealed that the STA was related to SCS, and the MS bar-graph of the STA is shown in Figure 4b.

Although a correlation between SCS and STA existed in *A. flavus*, the function of STA was not clear, so *stA* was deleted to explore the biofunction of STA. Deletion of *stA* was lethal to *A. flavus*; therefore, the ^*xylp*^*stA* strain was constructed to simulate *stA* knockout state. In Figure 4c-e, the ability of spore formation in ^*xylp*^*stA* was remarkably decreased compared to that in WT, when these media (YGT or GMM media) contained only glucose components (Figure 4c,d and Figure S3a,b). When both the WT and ^*xylP*^*stA* strains were grown on modified YGT or GMM media containing xylose or xylan (YXsT, YXnT, XsMM, or XnMM media), the growth and sporulation production of ^*xylP*^*stA* strain was recovered (Figure 4c,d and Figure S3a,b). The expression level of *stA* in the ^*xylP*^*stA* strain grown on XsMM or YXsT media, was considerably higher than that in the WT strain grown on XsMM or YXsT media (Figure 4e and Figure S3c). Taken together, ^*xylP*^*stA* was indeed a conditionally inducible strain based on the above analysis. Exploration was continued to validate the function of transferring the succinyl groups of STA. The ^*xylP*^*stA*-Δ*sucB*, which simulates Δ*sucB*Δ*stA*, was further constructed. The global succinylation levels in WT, Δ*sucB*, and ^*xylP*^*stA*-Δ*sucB* were measured using western blotting, and the result demonstrated that the global succinylation levels in ^*xylP*^*stA*-Δ*sucB* were significantly lower than those in Δ*sucB* (Figure 4f,g). Based on these results, we deduced that STA can transfer succinyl groups in *A. flavus*. Correspondingly, AFB1 accumulation in ^*xylP*^*stA* and ^*xylP*^*stA*-Δ*sucB* was remarkably decreased compared to that in WT and Δ*sucB* (Figure 4h,i). Totally, STA, with a succinyltransferase function, can change succinylation levels, which regulates AFB1 biosynthesis.

### The succinylation of AflM affects AFB1 and conidial formation

**Figure 5.**
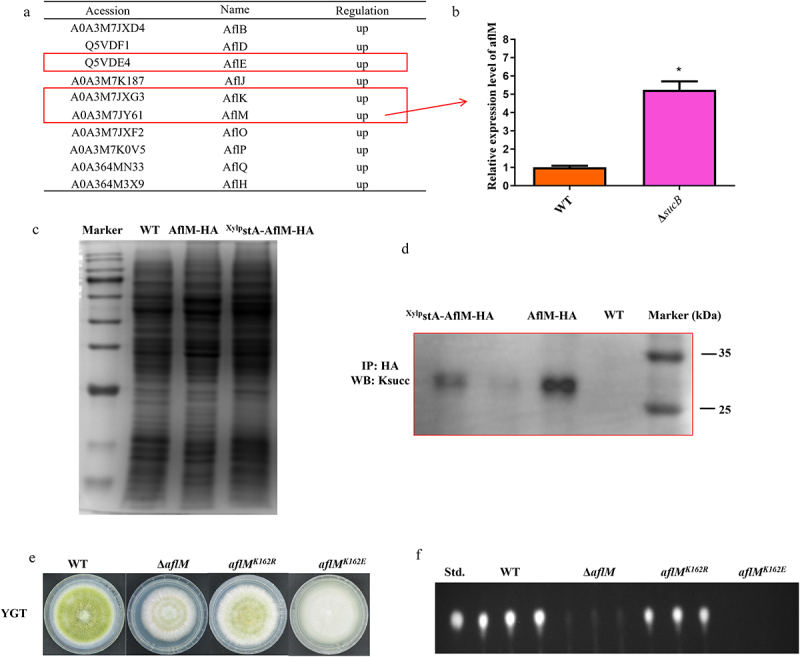
The influence of succinylated AflM in aflatoxin biosynthesis pathway on *A. flavus* phenotypes. (a) increased-abundant proteins of aflatoxin synthesis pathway screened by proteomics (Δ*sucB vs*. WT). Accession: protein ID in UniProt database. Red box: succinylated-proteins measured by LC-MS/MS [5]. (b) the expression level of *aflM* gene in Δ*sucB*. (c) the protein gel stained with coomassie brilliant blue (CBB) serves as a control for the amount of protein samples. (d) the immunoblot analysis of WT, AflM-HA and ^Xylp^stA-AflM-HA was performed with an anti-succinyllysine mouse monoclonal antibody. (e) vegetative growth of WT, Δ*aflM*, *aflM*^*K162E*^ and *aflM*^*K314R*^ strains on YGT media. (f) AFB1 production was measured by TLC after cultured in YES media at 29°C for 7 days.

The deletion of *sucB* led to the enrichment of DEPs in the aflatoxin biosynthesis pathway according to KEGG analysis, and most DEPs in this pathway were increased according to the above results. As shown in Figure 5a, abundances of several important aflatoxin-related proteins (AflB, AflD, AflE, AflJ, AflK, AflM, AflO, AflP, AflQ and AflH), were all increased in Δ*sucB* (Figure 5a). Especially, AflE, AflK, and AflM were also putative succinylated proteins according to Ren’s previous study [5]. There is an emphasis on AflM because it can generate the AFs biosynthesis- intermediate demethylsterigmatocystin (DMST). qRT-PCR was first applied to validate the expression level of *aflM*, and the expression of *aflM* in Δ*sucB* was upregulated compared to that in the WT (Figure 5b). Subsequently, *A. flavus* strains *AflM-HA* and ^*Xylp*^*stA-AflM-HA* were constructed. The immuno-precipitation assay confirmed that the AflM succinylation level in the ^*xylp*^*stA* strain was decreased compared to that in *AflM-HA* strain (Figure 5d), with a control of protein samples’ amount by Coomassie blue staining (Figure 5c). The above results indicate that STA is related to the succinylation of AflM. Additionally, AflM was succinylated in a normal biological environment, and the absence of SCS increased the expression level of AflM in the aflatoxin biosynthesis pathway, ultimately leading to an upregulation of aflatoxin biosynthesis in Δ*sucB*. Since AflM plays an important role in the biological synthesis of AFs, this study further investigated the biological functions of AflM and its succinylation through gene knockout and succinylated-site (K162) mutations. Figure S2b shows the sequencing results for the site mutants, demonstrating that *aflM*^*K162E*^ and *aflM*^*K162R*^ were successfully constructed in this study (Figure S2b). Furthermore, both knockout and *aflM*^*K162E*^ resulted in a significant decrease in *A. flavus* vegetative growth and sporulation formation (Figure 5e). With respect to AFB1 biological synthesis, it was found that AFB1 yield was significantly decreased in both Δ*aflM* and *aflM*^*K162E*^ strains (Figure 5f). For the other site-directed mutation aflM^K162R^, the result showed that the mutation of K162 to R can partially recover conidium formation and AFB1 yield compared to *aflM*^*K162E*^(Figure 5f). In summary, the above results indicated that STA can regulate the succinylation level of AflM, and succinylated AflM can affect the development and AFB1 biological synthesis of *A. flavus*.

### Differential proteomics analysis in (Δ*sucB* vs. WT) and (^xylp^stA-Δ*sucB* vs. Δ*sucB*)

Differential proteomics analysis in (Δ*sucB vs*. WT) and (^*xylp*^*stA*-Δ*sucB vs*. Δ*sucB*) was carried out, and quantitative proteomics results were evaluated by the distribution of abundance values between samples and principal component analysis (PCA). Figure S4a shows the integration diagram of the box-plot and violin-plot, which indicated that sample proteins were in reasonable abundance intervals (−5 to 5) and the intragroup consistency of biological samples was in the same group (Figure S4a). PCA suggested that different sample groups (WT, Δ*sucB*, and ^*xylp*^*stA*-Δ*sucB*) existed significant differences whereas sample differences in the same group were small, demonstrating that every sample met the requirements for proteomics analysis (Figure S4b).

In total, 1505 DEPs were identified in (Δ*sucB vs*. WT) (labeled Suc_vs_WT), of which 779 were increased and 726 were declined (Figure S5a). Similarly, 954 of 2094 DEPs were increased in (^*xylP*^*stA*-Δ*sucB vs*. Δ*sucB*) (labeled ST-Suc_vs_Suc), while the remaining 1140 were declined in (^*xylP*^*stA*-Δ*sucB vs*. Δ*sucB*) (Figure S5a). For the DEPs in (^*xylP*^*stA*-Δ*sucB vs*. WT), 1374 DEPs were identified in (^*xylP*^*stA*-Δ*sucB vs*. WT) (labeled as ST-Suc_vs_WT), of which 452 DEPs were increased-abundant proteins, whereas 922 were declined-abundant proteins (Figure S5a). The Venn diagram in Figure S5b shows that 243 DEPs among the 1505 DEPs were only presented in (Δ*sucB vs*. WT). In total, 290 DEPs among 1374 DEPs were only existed in (^*xylP*^*stA*-Δ*sucB vs* WT). Similarly, in (^*xylP*^*stA*-Δ*sucB vs* Δ*sucB*), only 443 DEPs were present among 2094 DEPs. In addition, 353 DEPs were found to co-exist in all DEPs of (Δ*sucB vs*. WT), (^*xylP*^*stA*-Δ*sucB vs*. WT), and (^*xylP*^*stA*-Δ*sucB vs*. Δ*sucB*) (Figure S5b).

All DEPs were utilized for the analysis of Gene ontology (GO) and KEGG pathways. GO analysis consists of three parts: molecular function (MF), biological process (BP), and cellular component (CC). As shown in Figure S5c,d, in the two comparison groups (Δ*sucB vs*. WT) and (^*xylP*^*stA*-Δ*sucB vs*. Δ*sucB*), the cellular anatomical entity was the most significantly enriched GO term in CC (Figure S5c,d). Catalytic activity, binding, and transporter activity were the most enriched terms in the MF (Figure S5c,d). Metabolic and cellular processes were the most significant GO terms for all BP (Figure S5c,d). In short, MF, BP, and CC of GO enrichment terms in (Δ*sucB vs*. WT), were the same as those of the GO terms in (^*xylP*^*stA*-Δ*sucB vs*. Δ*sucB*).

**Figure 6.**
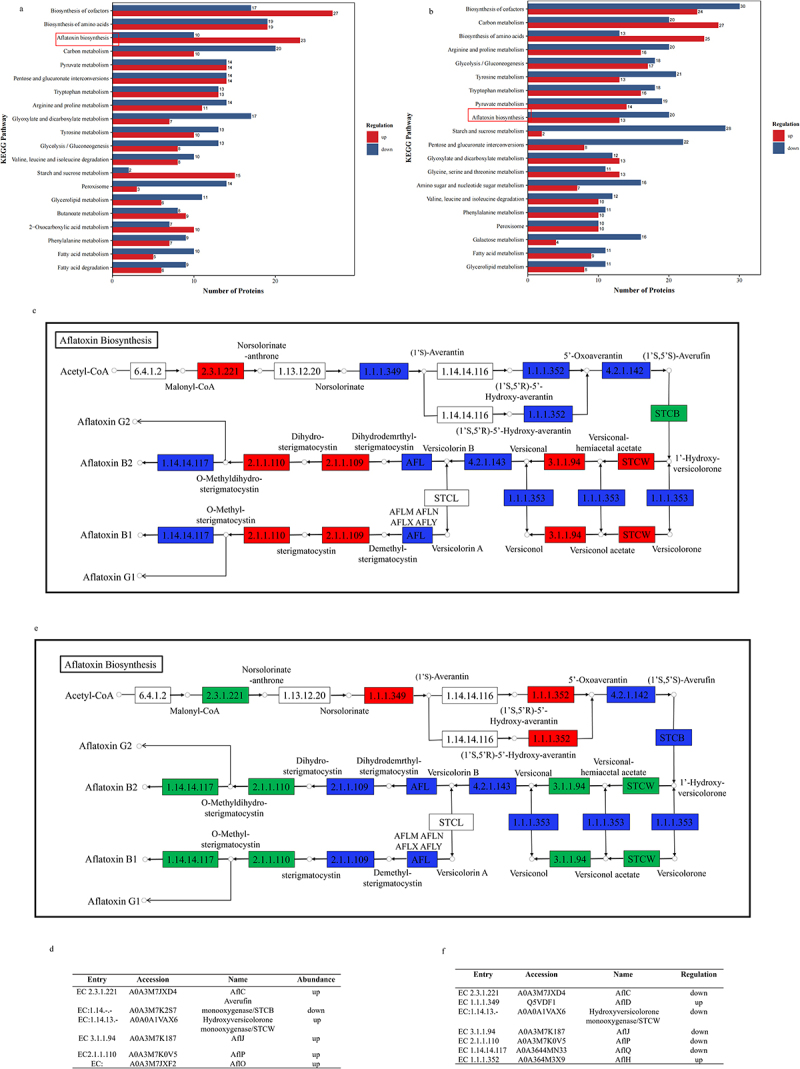
Differently abundant proteins in aflatoxin biosynthesis pathway (Δ*sucB vs*. WT; ^*xylp*^*stA*-Δ*sucB vs*. Δ*sucB*). (a) KEGG diagram of differential abundant proteins in (Δ*sucB vs*. WT). **(cb)** statistics of increased/decreased-abundant proteins in KEGG pathways (^*xylp*^*stA*-Δ*sucB vs*. Δ*sucB*). Different colors in the columns represent an increase or decrease in protein abundance. (c) differently abundant proteins marked in the aflatoxin pathway (Δ*sucB vs*. WT). Red: increased; green: decreased. (d) differently abundant proteins in (c). (e) differently abundant proteins marked in the aflatoxin pathway (^*xylp*^*stA*-Δ*sucB vs*. Δ*sucB*). Red: increased; green: decreased. (f) differently abundant proteins in (e).

Furthermore, the functional enrichment of the KEGG pathway in (Δ*sucB vs*. WT) was characterized. Most DEPs in (Δ*sucB vs*. WT) were enriched in biosynthesis of cofactors (44 DEPs), carbon metabolism (30 DEPs), biosynthesis of amino acids (38 DEPs), aflatoxin biosynthesis (33 DEPs), pentose and glucuronate interconversions (28 DEPs), tryptophan metabolism (26 DEPs), glycolysis/gluconeogenesis (21 DEPs), and other biological pathways (Figure 6a). In particular, 23 increased-abundant DEPs and 10 declined-abundant DEPs in the aflatoxin biosynthesis pathway were enriched, demonstrating that the deletion of SCS significantly affected the enzymes in the aflatoxin biosynthesis pathway (Figure 6a). Additionally, most DEPs in Δ*sucB* were enriched in the pathways of arginine and proline metabolism, tryptophan metabolism, and biosynthesis of amino acids, indicating that the absence of SCS perturbs most amino acid synthesis and partial amino acid metabolism (Figure 5a). Similarly, deletion of SCS caused changes in protein abundance in carbon metabolism (Figure 6a). KEGG pathway analysis in (Δ*sucB vs*. WT) indicated that the deletion of SCS broadly affected the aflatoxin biosynthesis pathway, amino acid biosynthesis/metabolism, sugar synthesis/metabolism, and even fatty acid synthesis/metabolism pathways (Figure 6a).

Functional enrichment of the KEGG pathway in (^*xylp*^*stA*-Δ*sucB vs*. Δ*sucB*) was characterized. As shown in Figure 6b, most DEPs were enriched in aflatoxin biosynthesis, biosynthesis of cofactors, starch and sucrose metabolism, pentose and glucuronide interconversions, tyrosine metabolism, fatty acid metabolism, glycolysis/gluconeogenesis, arginine and proline metabolism, and carbon metabolism pathways (Figure 6b). Collectively, elevated-abundant DEPs in Δ*sucB* were more than reduced-abundant DEPs, which demonstrated that the deletion of SCS caused the increase of most DEPs abundant levels. Reduced-abundant DEPs in (^*xylp*^*stA*-Δ*sucB vs*. Δ*sucB*) were more than elevated-abundant DEPs (Figure 6e,f), suggesting that low inducible expression of *stA* in Δ*sucB* caused the reduction of most protein abundances in enriched pathways.

### DEPs analysis of the aflatoxin biosynthesis pathway in WT, ΔsucB and ^xylP^stA-ΔsucB

KEGG analysis demonstrated that DEPs in mutant strains (Δ*sucB* and ^*xylP*^*stA*-Δ*sucB*) were enriched in the aflatoxin biosynthesis pathway; therefore, a deep analysis of DEPs in the aflatoxin biosynthesis pathway was performed. As shown in Figure 6c,d, the deletion of *sucB* resulted in increased abundances of AflC, AflJ, AflP, AflO, and hydroxyversicolorone monooxygenase (STCW), whereas it resulted in the decreased abundance of averufin monooxygenase (STCB) (Figure 6c). When *stA* in Δ*sucB* was expressed at low levels (^*xylp*^*stA*-Δ*sucB* grown on GMM can simulate the *stA* knockdown state in Δ*sucB)*, abundances of AflC, AflJ, AflP, STCW, and AflQ were reduced, whereas those of AflD and AflH were elevated (Figure 6f). These results indicated that STA positively regulates AflC, AflJ, AflP, and STCW in *A. flavus*. The low expression of *stA* in Δ*sucB* can lead to a decrease in aflatoxin accumulation. Furthermore, the abundance of AflQ, the enzyme directly responsible for AFB1 synthesis, was reduced in ^*xylP*^*stA*-Δ*sucB* mutant, which further clarified the reason for the decrease in AFB1 production (Figure 6f).

### Ach1 interacts with SCS to regulate AFB1 biosynthesis

**Figure 7.**
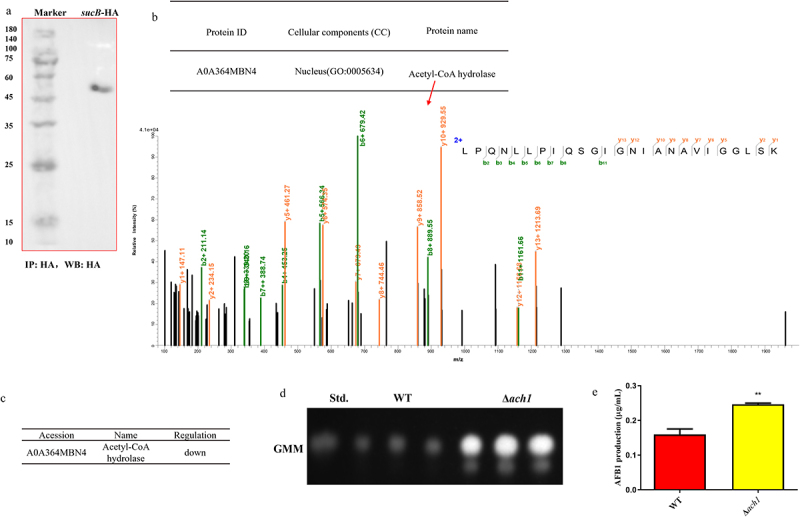
Acetyl-CoA hydrolase was the interaction protein of SCS by IP-MS and impacted AFB1 production. (a) IP enrichment result of *sucB*-HA. (b) mass spectrum of acetyl-CoA hydrolase that interacted with SCS in *A. flavus*. (c) acetyl-CoA hydrolase abundance was decreased in Δ*sucB* according to differential proteomics analysis in (Δ*sucB vs*. WT). (d) AFB1 production was measured by TLC after cultured in GMM media at 29°C for 7 days. (e) quantification of aflatoxin production when WT and Δ*ach* were cultured in GMM media. (**: *p* < 0.01).

Acetyl-CoA hydrolase (Ach1, accession number: G4B84_001523) catalyzes acetyl-CoA and succinate to form acetate and succinyl-CoA. The absence of SCS in *A. flavus* resulted in a low-abundance of Ach1 according to differential proteomics analysis (Figure 7c). This indicated that deletion of SCS led to the accumulation of succinyl-CoA, which led to the reduction of Ach1 abundance (Ach1 can catalyze acetyl-CoA to form succinyl-CoA) via feedback regulation.

The SCS with other proteins enriched by anti-HA antibody is shown in Figure 7a, and mass spectrometry identified that Ach1 interacted with SCS, as shown in Figure 7b. In total, Figure 7a-c demonstrated that abundance change of Ach1 was affected by SCS, which finally triggered the change of AFB1 production in Δ*sucB*. Furthermore, the influence of *A. flavus* Ach1 on AFB1 production was studied using *ach1* knockout. As shown in Figure 7d,e, AFB1 production in Δ*ach1* was significantly higher than that in the WT. These data clarified that Ach1 interacts with SCS, and the deletion of *sucB* would result in the reduction of Ach1 abundance, which further regulates downstream AFs biosynthesis.

### Untargeted metabolomics analysis of aflatoxin biosynthesis pathway in ΔsucB

In this study, differential metabolites in Δ*sucB* (Δ*sucB vs*. WT) were analyzed by untargeted metabolomics, and the results of metabolomics quality control (QC) are shown in Figure S6a-d. As shown in Figures S6a-d, the metabolomics detection system was credible, and the QC samples had good repeatability. Figure S6e shows the secondary differential metabolite cluster heat map, which indicated higher expression of differential metabolites (the gradient color: red) and lower expression of differential metabolites (the gradient color: blue) in (Δ*sucB vs*. WT). Figures S6f showed the correlation heatmap of secondary differential metabolites, which indicated that the relationship between the selected differential metabolites was biased toward a positive correlation.

**Figure 8.**
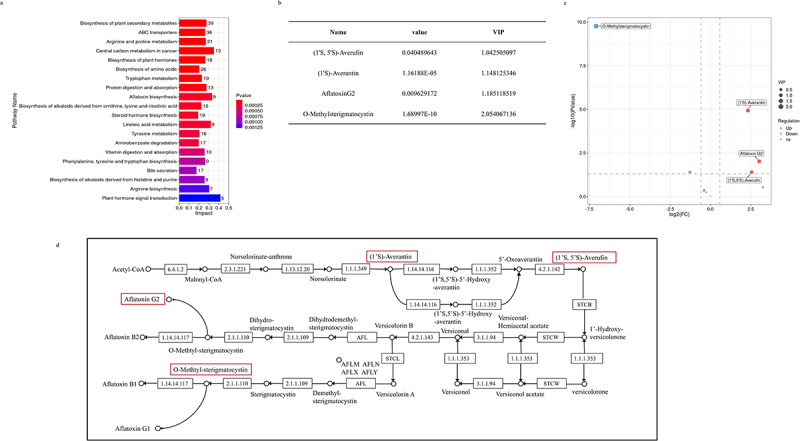
Analysis of differential metabolites in aflatoxin biosynthesis pathway (Δ*sucB vs*. WT). (a) KEGG analysis of differential metabolites in *A. flavus* (Δ*sucB vs*. WT). (b) different metabolites of aflatoxin biosynthesis pathway in *A. flavus*. (c) volcano plot of nine differential metabolites in aflatoxin biosynthesis pathway. (d) four differential metabolites in aflatoxin biosynthesis pathway (*https://www.kegg.jp/entry/map00254*).

Subsequently, all metabolites in (Δ*sucB vs*. WT) were enriched in Figure 8a’s pathways. As shown in Figure 8a, most metabolites were involved in the biosynthesis of amino acids, phenylalanine, tyrosine, and tryptophan, arginine and proline metabolism, tyrosine metabolism, biosynthesis of plant secondary metabolites, and aflatoxin biosynthesis (Figure 8a). In aflatoxin biosynthesis pathway, nine metabolites (averantin, aflatoxin B2, aflatoxin G1, aflatoxin G2, (1’S,5’S)-averufin, (1’S,5’R)-5’-hydroxyaverantin, (1’S)-averantin, dihydro-O-methylsterigmatocystin, and O-methylsterigmatocystin) were enriched. Based on the screening criteria for differential metabolites (variable importance in the projection (VIP) value >1 and simultaneous *p* value < 0.05), four differential metabolites in aflatoxin biosynthesis were selected: (1’S,5’S)-averufin, (1’S)-averantin, O-methylsterigmatocystin, and AFG2 (Figure 8b). As shown in Figure 8c, (1’S,5’S)-averufin, (1’S)-averantin, and AFG2 were upregulated compared to the WT, but O-methylsterigmatocystin was downregulated compared to the WT. These upregulated and downregulated AF-related metabolites explain the reasons for the increasing in AFB1 yield. In addition, O-methylsterigmatocystin was rapidly converted to AFB1 caused by the downregulation of O-methylsterigmatocystin, so AFB1 yield in Δ*sucB* was increased compared to AFB1 in WT (Figure 8c). Figure 8d shows the detailed positions of AF-related metabolites in the aflatoxin biosynthesis pathway.

### Analysis of metabolic pathways and differential metabolites in *A. flavus* growth development and sclerotial formation

**Figure 9.**
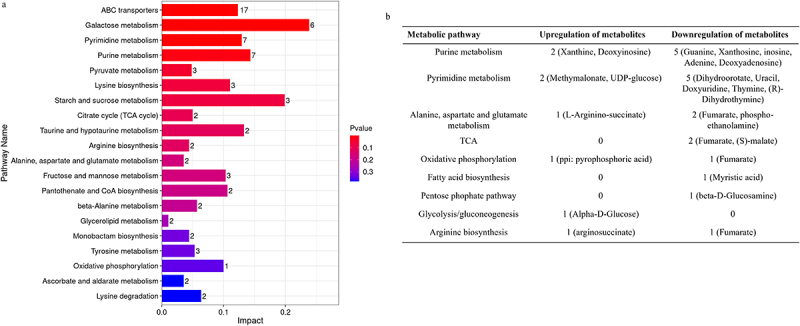
KEGG pathways and differential metabolites involved in *A. flavus* development and sclerotial formation. (a) metabolic pathway enrichment map (WT *vs*. Δ*sucB*). (b) differential metabolites (Δ*sucB vs*. WT).

In addition to the effect of SCS on aflatoxin biosynthesis described above, we also applied the untargeted metabolome to classify the basic metabolic pathways, and finally conducted enrichment analysis for these metabolites. As shown in Figure 9a, these metabolites were mainly enriched in galactose, pyrimidines, purines, citric acid cycle, oxidative phosphorylation, pyruvate, glycine, aspartate, and glutamate metabolism. Next, we focused on the arginine synthesis pathway, pyrimidine metabolism, purine metabolism, citric acid cycle, oxidative phosphorylation, glycolysis/gluconeogenesis pathway, fatty acid synthesis, and glycine and aspartate metabolisms to screen out the differences in these pathways.

As reported in the literature by Valdet Uka *et al*., Alanine, aspartate, and glutamate metabolism are related to sclerotial development, and fatty acid synthesis is related to aflatoxin production [27]. In this study, the metabolite L-arginino-succinate in the (Alanine, aspartate, and glutamate metabolism) pathway was upregulated (Figure 9b), while fumarate and phosphoethanolamine were downregulated (Figure 9b). These results indicate that SCS knockdown might have an impact on sclerotial formation. In addition, basic metabolic pathway analysis found that α-D-glucose was upregulated in glycolysis and gluconeogenesis pathways (Figure 9b), uridine 5’-(dihydrogen diphosphate)-glucose UDP glucose (nucleotide sugar, was a glucose donor) was upregulated in pyrimidine metabolism (Figure 9b), malate and fumarate were downregulated in the TCA cycle (Figure 9b), β-D-glucosamine in the pentose phosphate pathway was downregulated (Figure 9b), myristic acid (also involved in aflatoxin biosynthesis) was downregulated in the fatty acid synthesis pathway (Figure 9b), and fumarate in oxidative phosphorylation was downregulated, whereas pyrophosphoric acid was upregulated (Figure 9b). In conclusion, many factors affect the growth and development of *A. flavus*, but metabolite changes in these basic pathways have a significant impact on *A. flavus* growth and development.

## Discussion

SCS, a β-subunit of ATP-specific succinyl-CoA synthetase, is related to succinyl-CoA formation. Succinyl-CoA, as a succinyl group donor, inevitably affects the succinylation of total protein in *A. flavus*. As Ren *et al*. reported, protein succinylation is universal in *A. flavus* [5], which is involved in phenotypic changes of aflatoxin biosynthesis, conidiation, and sclerotium formation. In this study, when SCS was disabled in *A. flavus*, the succinyl-CoA content in Δ*sucB* was upregulated, and the global succinylation level of Δ*sucB* mutant was increased when compared to WT. In yeast, disruption of succinyl-CoA ligase can increase global protein succinylation [4], which was similar to the results from this study in *A. flavus*. Obviously, these results uncovered that SCS plays an important role in the regulation of global succinylation levels. Furthermore, AFB1 production from Δ*sucB* mutant was measured, and the result indicated that AFB1 accumulation was increased in Δ*sucB* mutant. Consequently, it can be concluded that the deletion of *sucB* in *A. flavus* causes an increase in global protein succinylation based on the accumulation of succinyl-CoA in Δ*sucB*, which finally influences aflatoxin B1 yields and other phenotypic changes (*i.e., A. flavus* conidiation/sclerotium formation). Lancaster *et al*. demonstrated that loss of succinyl-CoA synthetase (SCS) in mouse forebrain resulted in accumulation of succinyl-CoA and causing global protein hypersuccinylation. Especially, the succinylation of mitochondrial pathways proteins and five histone proteins (H1, H2A, H2B, H3, and H4) in mutant cortex can further impact neuron development and neuronal function [14]. Tong *et al*. found that the S79 site of SUCLA2 (succinyl-CoA synthase ADP-forming subunit β) is phosphorylated by p38 MAPK under oxidative stress, which causes its dissociation from kidney-type glutaminase (GLS), resulting in enhanced GLS K311 succinylation, oligomerization, and activity. In particular, the activated GLS can promote the mice’s tumor cell survival and growth by increasing the production of glutathione and nicotinamide adenine dinucleotide phosphate (NADPH) [28]. The above explorations indicate that succinyl-CoA synthetase regulates the succinylation of different proteins/enzymes in different species and causes a series of pathological and fungal phenotypic changes.

Subsequently, we identified a succinylated protein AflM which triggered by SCS deficiency and clarified its biofunctions. As shown in Figure 5, by differential proteomics analysis of (Δ*sucB vs* WT), we found that most of the aflatoxin biosynthesis-related DEPs in Δ*sucB* were upregulated. In particular, a putative succinylated AflM detected by LC-MS/MS was considered in this study [5]. Western blotting and Co-IP experiments were performed to validate the succinylation of AflM, and the results demonstrated that AflM is a succinylated protein under normal physical conditions. The influence of AflM succinylation on AFB1 biosynthesis was determined by succinylated-site mutations in AflM. The result showed that *aflM*^*K162E*^ had the obvious effect on AFB1 production, suggesting that succinylation of AflM has an important impact on AFB1 biosynthesis.

It is widely known that α-ketoglutarate dehydrogenase (α-KGDH) complex usually comprises three components: oxoglutarate dehydrogenase (OGDH), dihydrolipoyl succinyltransferase (DLST), and dihydrolipoyl dehydrogenase (DLD). In this study, we demonstrated that STA (a dihydrolipoyl succinyltransferase in *A. flavus*) is a crucial succinyllysine transferase based on the measurement of global succinylation levels in Δ*sucB* and ^*xylp*^*stA*-Δ*sucB* mutants. Furthermore, succinylation levels of AflM-HA and ^xylp^StA-AflM-HA were detected to clarify the influence of STA on the AflM’s succinylation, and the results showed that AflM’s succinylation was down-regulated when *stA* was low expressed (Figure 5c,d). The above demonstrated that AflM’s succinylation is regulated by STA, and STA is relevant to SCS according to IP-MS and western blot experiments (Figure 4a,b,f,g). In summary, STA functions as a succinyltransferase and affects global protein succinylation levels by coupling with SCS, ultimately affecting AFB1 production in *A. flavus*. Wang *et al*. reported that lysine acetyltransferase 2A (KAT2A, a member of the GCN5-related N-acetyltransferase superfamily) would together with the OGDH of α-KGDH complex to transfer the succinyl group to histone H3K79, and the succinylation of H3K79 can promote brain tumor growth in mice [29]. In addition, KAT2A did not interact with the DLST-DLD complex to exert its corresponding functions when OGDH was depleted [29]. Above results from Wang *et al*. are similar to our studies in *A. flavus.*

*A*. *flavus* Ach1 interacted with SCS, and its abundance was declined in Δ*sucB* based on differential proteomics analysis (Δ*sucB* vs WT), suggesting that Ach1 is a relevant enzyme in the SCS regulation pathway. To our knowledge, the function of Ach1 in *A. flavus* is consistent with that in *S. cerevisiae*’s Ach1 [30,31]. In *S. cerevisiae*, Ach1 in the mitochondria is an acetate: succinate CoA transferase, which transfers CoASH moiety from succinyl-CoA to acetate and finally leads to the formation of acetyl-CoA and succinate [32]. In *A. flavus*, Ach1 expression was downregulated in Δ*sucB*. The function of Ach1 in *A. flavus* was characterized by the deletion of the *ach1* gene, showing that deletion of *ach1* would increase AFB1 production. Fleck and Brock also clarified that Ach1 (CoaT in *A. nidulans*) functions in the detoxification of *A. nidulans* and found that CoaT from *A. nidulans* was highly similar to Acu-8 of *Neurospora crassa* and Ach1p of *S. cerevisiae* [33]. Additionally, CoaT was considered to be a CoA-transferase with minor hydrolase activity, and can transfer the CoASH moiety from toxic propionyl-CoA to acetate, which is effective in reducing the amount of toxic propionyl-CoA in *A. nidulans* [33]. These results revealed that Ach1 can affect *Aspergillus* virulence (*A. flavus* or *A. nidulans*).

## Conclusion

This study explored the effects of SCS on the development and aflatoxin production of *A. flavus* by comparative proteomics and metabonomics. Depending on the increase of succinyl-CoA content and STA transferring succinyl group function, the deficiency of SCS (Succinyl-CoA synthase ADP-forming subunit β) can improve the succinylation level of global protein and increase AFB1 production in *A. flavus*. Furthermore, proteome analysis uncovered that abundances of several aflatoxin-related proteins (AflB, AflD, AflE, AflJ, AflK, AflM, AflO, AflP, AflQ and AflH) were elevated in Δ*sucB*. By the detection of IP-MS and Co-IP, the AflM among these proteins was a succinylated protein, and its succinylation level was regulated by STA. Additionally, SCS also correlated with STA and Ach1, and they co-regulated the AFB1 biosynthesis through succinylation modification of aflatoxin-related protein. Collectively, we deduced a specific pathway associated with aflatoxin biosynthesis: Ach1/STA-SCS-succinyl-CoA-succinylated AflM-AFB1 biosynthesis.

## Materials and methods

### Strains and culture conditions

*A*. *flavus* NRRL3357 was used as the wild-type (WT) strain in this study, and TXZ 21.3 with auxotrophic markers (*pyrG*-/*argB*-) was used as the original strain to construct mutants (deleted or site-directed mutants). All strains were activated on PDA (39 g/L potato dextrose agar, BD Difco, Franklin, NJ, USA) at 37°C for 4 days and grown on four replicate plates in the dark. The experiments were repeated at least thrice. The strains used in this study are listed in Table 1.

**Table 1.** Strains used in this study.

Strain	Genotype description	Source
NRRL3357	*A. flavus* WT	Nancy P. Keller lab
TJES19.1	*pyrG1*, Δ*ku70*	[34]
TXZ 21.3	*pyrG1*, Δ*ku70*, Δ*argB*	[34]
TJES 20.1	*pyrG1:pyrG*, Δ*ku70*, Δ*argB*	[34]
Δ*sucB*	*pyrG1*, Δ*ku70*, Δ*sucB: pyrG*	This study
Δ*sucB-*com	*pyrG1*, Δ*ku70*, Δ*sucB: pyrG:sucB*	This study
Δ*aflM*	*pyrG1*, Δ*ku70*, Δ*aflM: pyrG*	This study
*aflM*^*K162E*^	*pyrG1*, Δ*ku70, aflM*^*K162E*^:: *pyrG*	This study
*aflM*^*K162R*^	*pyrG1*, Δ*ku70, aflM*^*K162R*^:: *pyrG*	This study
Δ*ach1*	*pyrG1*, Δ*ku70, Δach1:pyrG*	This study
^*XylP*^*stA*	*pyrG1*, Δ*ku70*, Δ*argB, argB: xylP:stA*	This study
^*XylP*^*stA-*Δ*sucB*	*pyrG1*, Δ*ku70*, Δ*argB*, *argB::xylP:stA*, Δ*sucB::pyrG*	This study

### Sequence analysis and construction of the phylogenetic tree

The amino acid sequence of succinyl-CoA synthetase subunit β (SCS,G4B84_0111276) was downloaded from the NCBI database (*https://www.ncbi.nlm.nih.gov*). Other SCS homologs in different species were obtained using the BLAST tool in the NCBI. Phylogenetic trees were constructed using DNAMAN and MAGA 5.1 softwares [35]. Domain analysis was performed using SMART (*http://smart.emblheidelberg.de/smart/set_mode.cgi?NORMAL=1*), and the protein domain diagrams of corresponding species were drawn using DOG 2.0 software [36].

### Construction of mutant strains

All strains were constructed based on the principle of homologous recombination, and the auxotrophic strain TJES19.1 was served as the starting strain. To construct the *sucB* gene deletion strain (Δ*sucB*), *pyrG* from *A. fumigatus* was inserted into *A. flavus* to replace its *sucB* gene. The construction of Δ*aflM* and Δ*ach1* followed the same principle as Δ*sucB*. Construction of the complementary strain (Δ*sucB-com*) required two steps. In the first step, the 5’UTR-*sucB* ORF-3’UTR fusion fragment was reinserted into the protoplasts of the Δ*sucB* strain, and 5-Fluoroorotic acid was used for resistance screening in the first step. In the second step, the 5’ORF-*pyrG-*3’UTR fragment was transformed into the protoplasts of the complementary strain obtained in the first step. To construct HA-tagged mutants, the *aflM* gene fragment, *pyrG* with 3×HA tag, and the downstream region of *aflM* were fused via PCR. The fusion fragment (*aflM* ORF-3×HA-*pyrG-*3’UTR of aflM) was transformed into protoplasts of *A. flavu*s TJES19.1. The *sucB* −3×HA-*pyrG* strain was constructed using the same method. To construct ^*XylP*^*stA* (mimicking the *stA* deletion status), four fragments containing the upstream fragment of the *stA* gene, *pyrG*, xylP (xylose-inducible promoter), and the ORF fragment of the *stA* gene were overlapped using fusion PCR. The fusion fragment was transformed into the TJES19.1 protoplast. For the construction of ^*XylP*^*stA-*Δ*sucB*, TXZ 21.3 with two auxotrophic *pyrG* and *argB* was used as the original strain for construction. All primers used are listed in **Table S1**.

### Phenotype analysis

For spore analysis, the strains were grown in PDA medium for 5 days in a dark incubator at 37°C. After dilution to 10^6^ spores/mL, 1 μL of fungal suspension was inoculated onto GMM, YGT, or YES plates. Spores were collected and counted using a hemocytometer. For sclerotia analysis, 1 μL of spore suspension (10^6^ spores/mL) was inoculated into petri dishes containing CM medium. The dishes were sealed with Parafilm and incubated in a dark incubator at 37°C for 7 days. Petri dishes were punched, and sclerotia were counted [7].

### Aflatoxins analysis

About 50 μL of spore suspension (10^6^ spores/mL) was inoculated into the YES/GMM liquid medium and incubated in a dark chamber at 29°C for 7 days. AFB1 extraction and detection were performed as previously described [37–39].

### Immunoprecipitation and LC-MS/MS analysis

About 500 μL of fungal suspension (10^6^ spores/mL of WT, *sucB*-HA, Δ*sucB*, ^*xylP*^*stA*-Δ*sucB*, ^*xylp*^*stA-aflM*-HA, or *aflM*-HA) was inoculated into 150 mL of GMM liquid medium and incubated at 37°C for 2 days. Total protein extraction from mycelia of mutant strains was performed as previously described methods [7,40]. Anti-HA magnetic beads were used for binding the target protein (*sucB*-HA, *aflM*-HA, ^*xylp*^*stA-aflM*-HA, or WT). The anti-HA antibody (Sigma-Aldrich) was applied for immunoblot analysis, as reported by Xie *et al*. [7]. Additionally, an anti-succinyllysine mouse monoclonal antibody (anti-succinyllysine mouse mAb, Hangzhou Jingjie Biotechnology Co., Ltd., China) was used to detect global succinylation levels of strain (Δ*sucB*, ^*xylP*^*stA*-Δ*sucB*, or WT) and AflM succinylation in mutant (WT, *aflM*-HA, or ^*xylp*^*stA-aflM*-HA). The protein solution from the *sucB*-HA strain was concentrated to a volume of less than 4 mL using a Millipore protein concentration tube (50 mL capacity). Subsequently, the concentrated protein solution was separated on SDS-PAGE gels and stained with Coomassie Brilliant Blue R250. The stained gels were cut off and subjected to LC-MS/MS analysis (Beijing Allwegene Technology Co., Ltd). All proteins were measured using the Goldband 3-color Regular Range Protein Marker (10–180 kDa, 20351ES72, Yeasen).

### Metabolomic and proteomics analysis

About 10 μL of spores suspension (10^6^ spores/mL) from WT or Δ*sucB* was incubated in GMM at 29°C in the dark for 7 days. Mycelia were frozen in liquid nitrogen, collected, and stored at −80°C. Samples were extracted with methanol, centrifuged, concentrated with 2-chloro-L-phenylalanine, filtered through a 0.22 μm membrane, and analyzed by LC-MS. Chromatography was conducted using a Thermo Vanquish UHPLC with an ACQUITY UPLC® HSS T3 column at 0.3 mL/min, 40°C, and 2 μL injection (Zelena *et al*. [41]). Mass spectrometry was performed using a Thermo Q Exactive mass spectrometer with an ESI source in positive and negative ion modes (Want *et al*., [42]). Proteomic analysis and sample processing were performed according to the methods of Wuhan Metware Biotechnology Co., Ltd. (www.metware.cn).

### Detection of succinyl-CoA content

About 100 μL of spores suspension (10^6^ spores/mL) from each strain were inoculated into 150 mL of YES liquid medium. After 2 days of incubation at 37°C, 180 rpm, the mycelia were filtered, frozen in liquid nitrogen, and powdered. The powder was weighed and aliquoted into 100 mg EP tubes. Using the Succinyl-CoA Detection Kit (Shanghai Shifeng, Sfk5693), reagents were added to determine the succinyl-CoA content.

### Quantitative real-time PCR assay

Quantitative Real-time PCR (qRT-PCR) of the WT and mutant strains was performed as previously described [7]. Data were collected, and relative gene expression levels were calculated using the 2^−ΔΔCt^ method. The specific reaction protocol followed the instructions for the TransScript® One-Step gDNA Removal and cDNA Synthesis SuperMix.

### Statistical analysis

All experiments were repeated three times, and the data are expressed as mean ± standard deviation. Statistical and significance analyses were performed using GraphPad Prism 5.01 software, with significance defined as *p* < 0.05. Significant differences were determined using one-way ANOVA. When performing multiple comparisons, Tukey’s multiple comparison test was used to analyze significance among the groups.

## Supplementary Material

complementary figures.docx

Figure S1.tif

Figure S2.tif

Figure S3.tif

Figure S6.tif

Figure S4.tif

Figure S5.tif

## Data Availability

The datasets underlying conclusions of this article and its supplementary files have been deposited in the 4TU.ResearchData (https://data.4tu.nl/my/datasets), which are openly available at 10.4121/b4ac6121-29d9-4cbc-b986-cd44450ac0db.

## Significance statement

The harm of *A. flavus* and its metabolite AFs (AFB1) on humans, livestock, and crops have been a topic of concern, as studying the functions and regulatory mechanisms of *A. flavus* genes can better reveal principles of *A. flavus* and AFs contaminating organisms. This study aimed to identify the proteins that regulate aflatoxin synthesis through SCS. Bio-informatics analysis of proteomics and metabolomics, as well as using the technologies of co-IP and western blotting, revealed an important regulatory pathway: Ach1/STA-SCS-succinylated AflM for AFB1 biosynthesis, and the effect of succinylated aflatoxin protein AflM on AFB1 production. Collectively, this study provides a theoretical basis for the prevention and control of *A. flavus*.
