# Supplementary material for: STA regulates succinylated AflM triggered by SCS to contribute to aflatoxin biosynthesis through the Ach1
Source: Virulence. 2025 Jul 18;16(1):2532812. doi: 10.1080/21505594.2025.2532812 (PMC12279275; doi:10.1080/21505594.2025.2532812)
Supplement: complementary figures.docx [file KVIR_A_2532812_SM9894.docx]

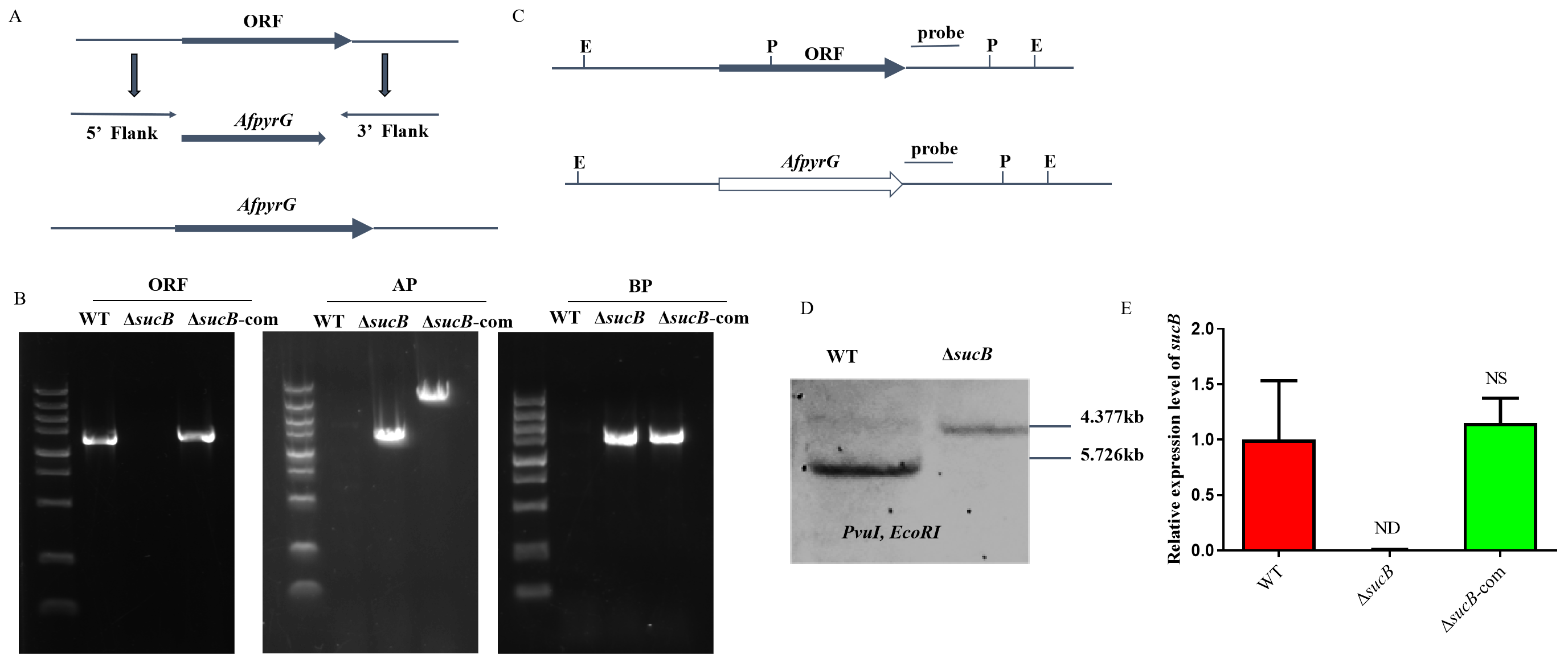


**Figure S1 Construction and verification of succinyl-CoA synthetase deletion strain.** (A) Construction schematic of succinyl-CoA synthetase deletion strain. (B) PCR verification of WT and deletion mutant. (C) Schematic diagram of southern blot. Cleavage positions digested by enzymes *PvuI* (P) and *EcoRI* (E) were showed on the WT and Δ*sucB.* The probe to hybridize the genomic DNA of WT and Δ*sucB* after enzyme digestion was also marked. (D) Southern blot hybridization analysis in WT and Δ*sucB*. (E) Expression level of *sucB* in WT, Δ*sucB* and Δ*sucB*-com. NS: not significan; ND: not detection.


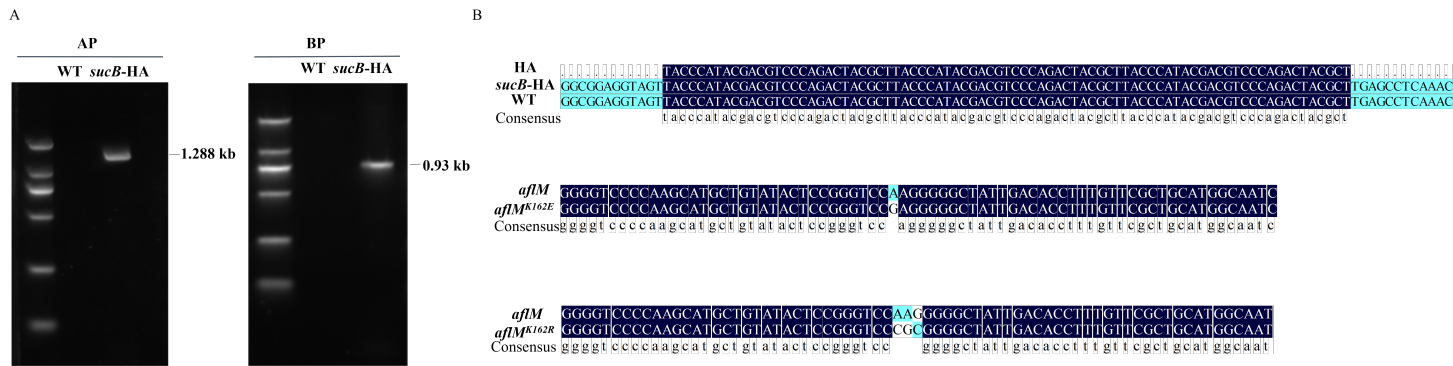


**Figure S2** **Validation of *sucB*-HA, *aflM^K162E^*, and *aflM^K162R^*.** (A) PCR verification of *sucB*-HA. (B) The sequencing results of *sucB*-HA, *aflM^K162E^*, and *aflM^K162R^* were blast by DNAMAN software.


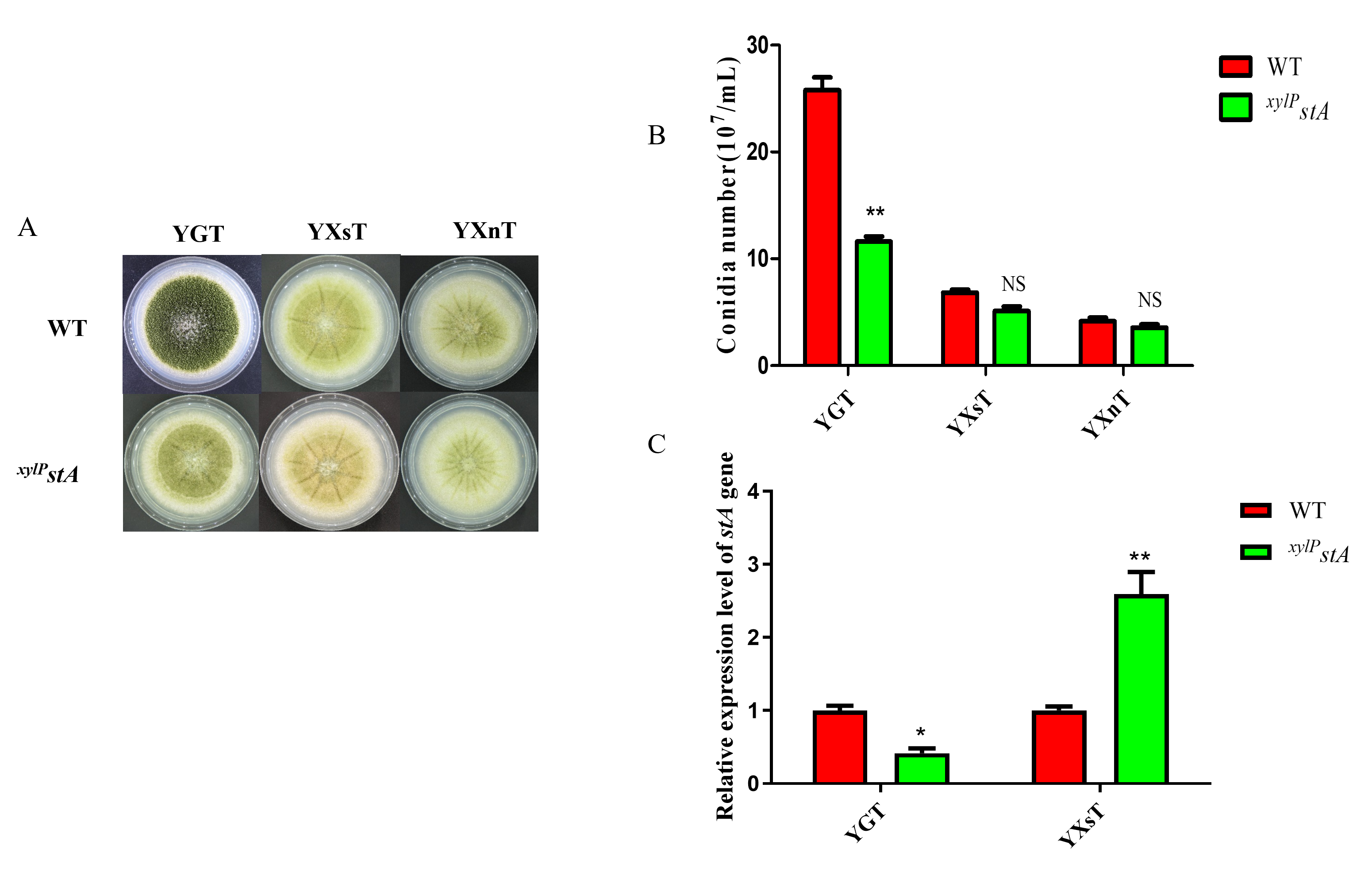


**Figure S3 Vegetative growth and conidiation of WT and *^xylP^stA* on differient media.** (A) Colonies formed by WT and *^xylP^stA* on YGT, YXsT, and YXnT agar plates. (B) Conidia number of WT and *^xylP^stA* strains grown on YGT, YXsT, and YXnT. (C) Relative expression level of *stA* gene in WT and *^xylP^stA* grown on YGT and YXnT agar plates (*: *p*<0.05, **: *p*< 0.01, NS: not significant).


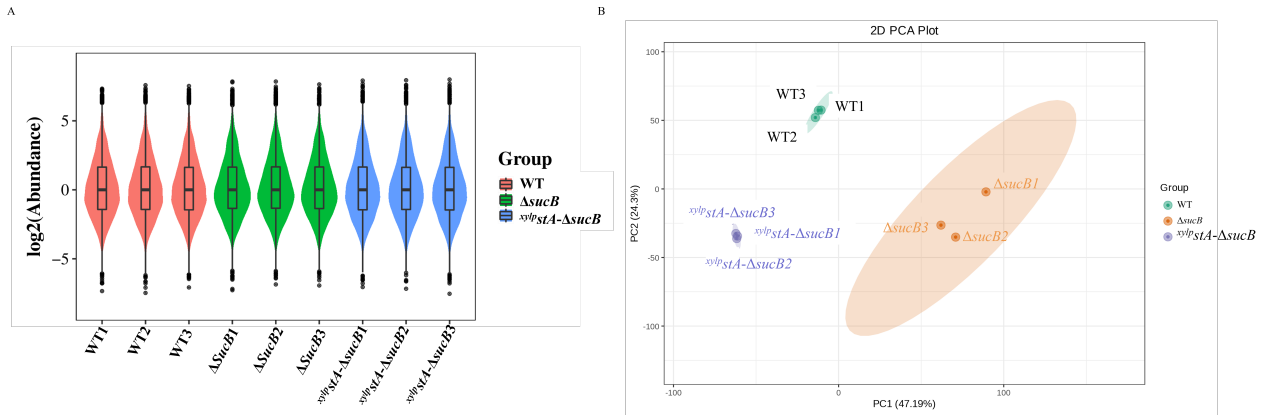


**Figure S4** **Abundance value distribution map and principal component analysis (PCA) from WT, Δ*sucB*, and *^xylP^stA*-Δ*sucB* grown on GMM media.** (A) Abundance value distribution map of WT, Δ*sucB*, and *^xylP^stA*-Δ*sucB*. (B) PCA plot of Measurement of WT, Δ*sucB*, and *^xylP^stA*-Δ*sucB*. (PCA, Principal Component Analysis)


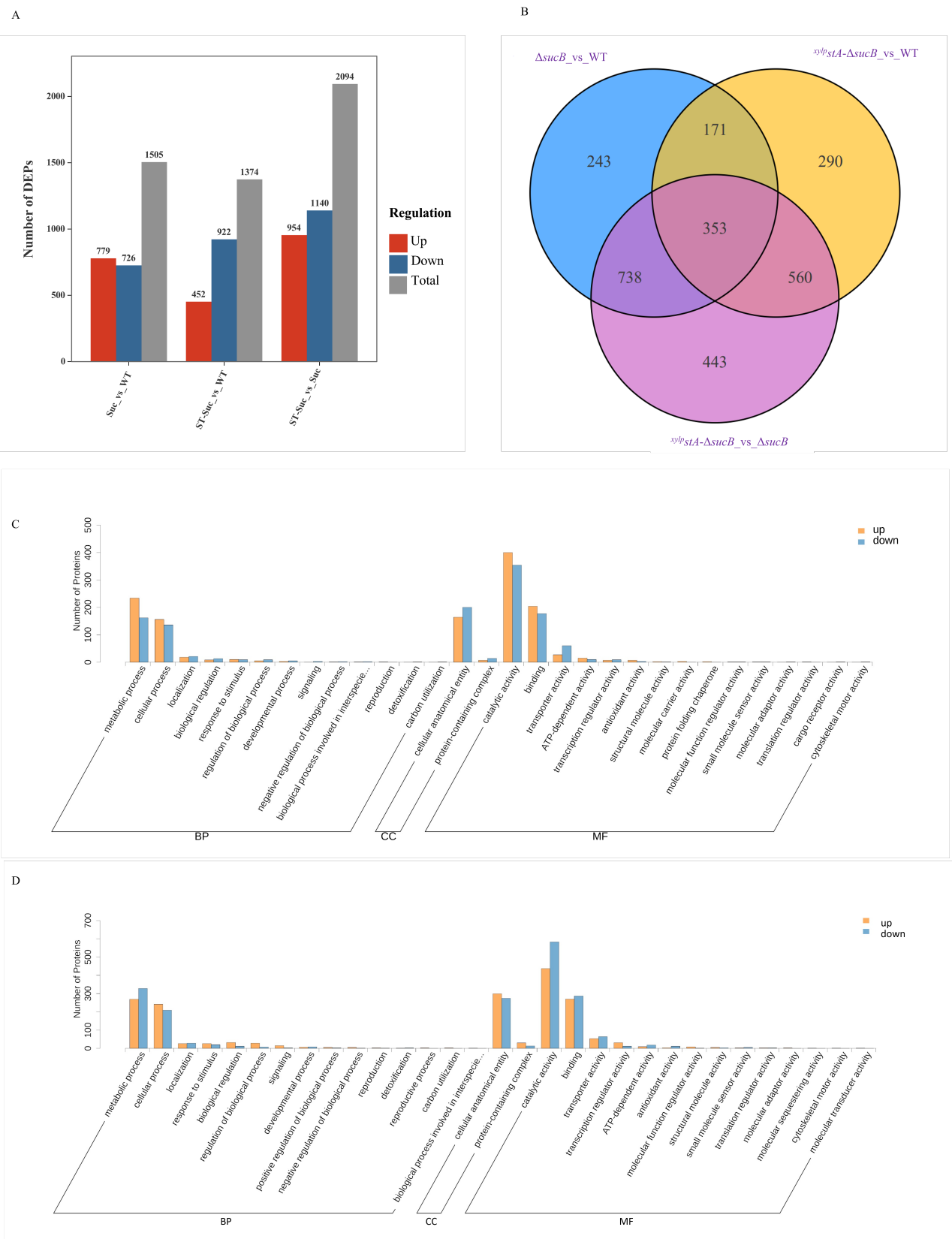


**Figure S5** **Differential analysis results statistics, differential protein grouping Venn diagram and GO analysis of differential protiens.** (A) Statistical chart of difference analysis results. (B) Differential protein grouping Venn diagram (WT, Δ *sucB* and *^xylP^stA*- Δs*ucB*). (C) GO analysis of differential proteins in (WT *vs.* Δ*sucB*). (D) GO analysis of differential proteins in (*^xylP^stA*- Δs*ucB* *vs.* Δ*sucB*).


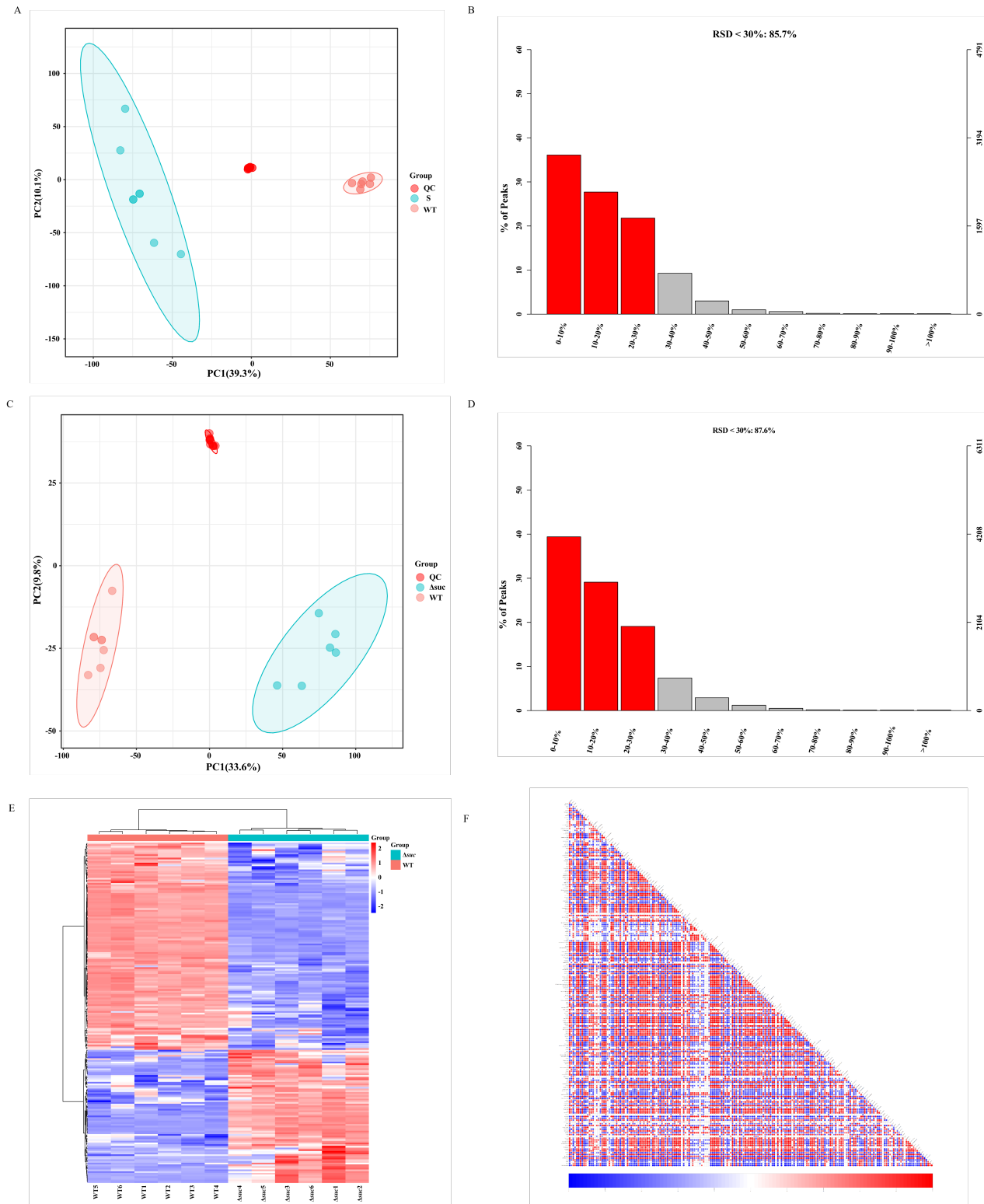


**Figure S6** **Quality control (QC) diagrams of positive/negative ions and correlation heatmaps of secondary differential metabolites**. (A) In negative ion mode, PCA (Principal Component Analysis) diagram of WT, Δ*sucB*, and QC samples. (B) In negative ion mode, the RSD (Relative Standard Deviation) of QC samples. (C-D) QC diagrams in positive ion mode. Red point represents QC samples, others represent WT and Δ*sucB*. (E) Cluster heatmap of secondary differential metabolites. Column represents samples WT1-6, and Δsuc1-6. Row represents differential metabolites. The cluster tree on the left is the differential metabolite cluster tree, and the top is the sample cluster tree. (F) Correlation heatmap of secondary differential metabolites. Oblique and oblique ordinate represent names of differential metabolites, and color representing correlation, red indicating positive correlation, and blue indicating negative correlation. The darker the color, the higher the correlation.
