# Supplementary figures and images for: STA regulates succinylated AflM triggered by SCS to contribute to aflatoxin biosynthesis through the Ach1
Source: Virulence. 2025 Jul 18;16(1):2532812. doi: 10.1080/21505594.2025.2532812 (PMC12279275; doi:10.1080/21505594.2025.2532812)

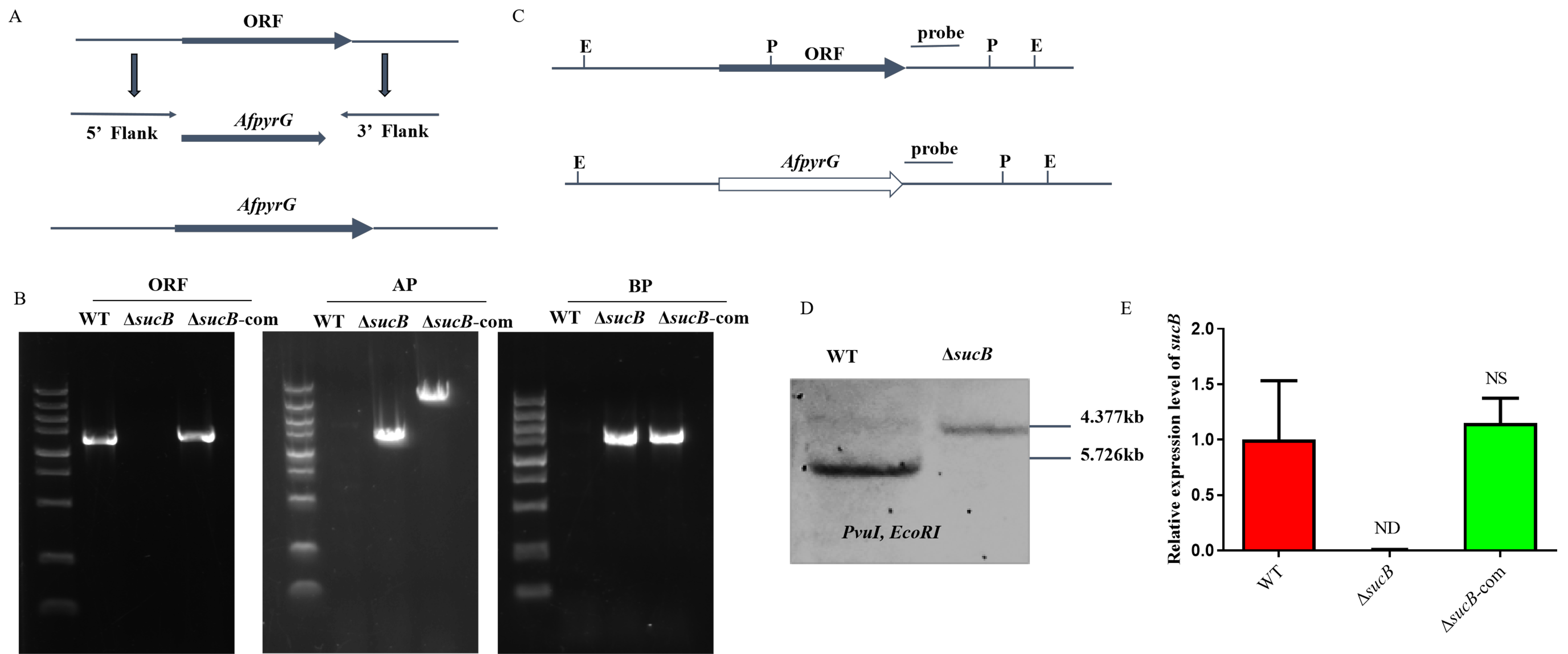

Supplement: Figure S1.tif [file KVIR_A_2532812_SM9893.tif]

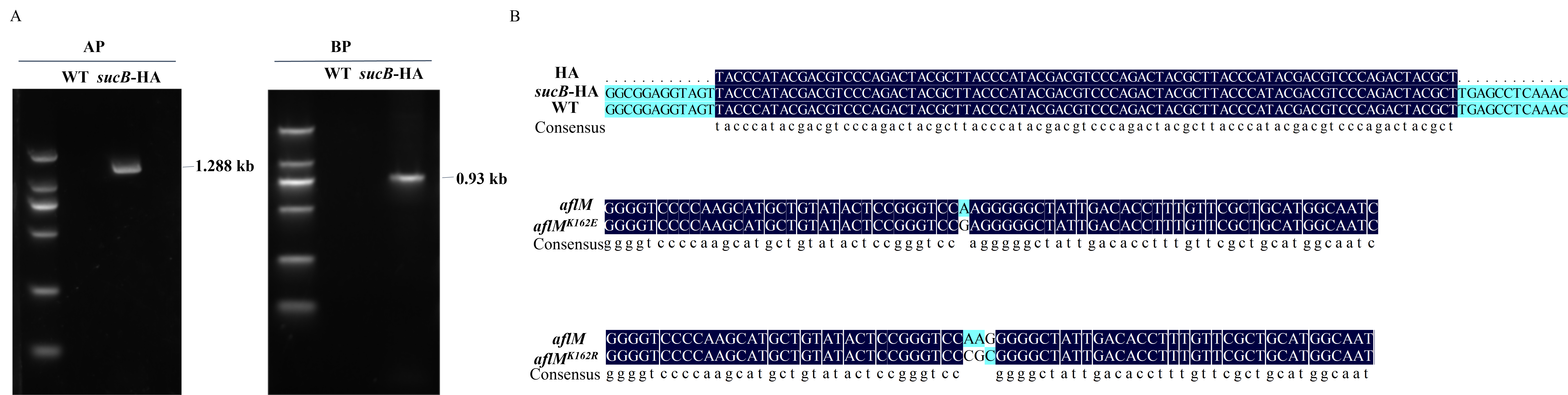

Supplement: Figure S2.tif [file KVIR_A_2532812_SM9892.tif]

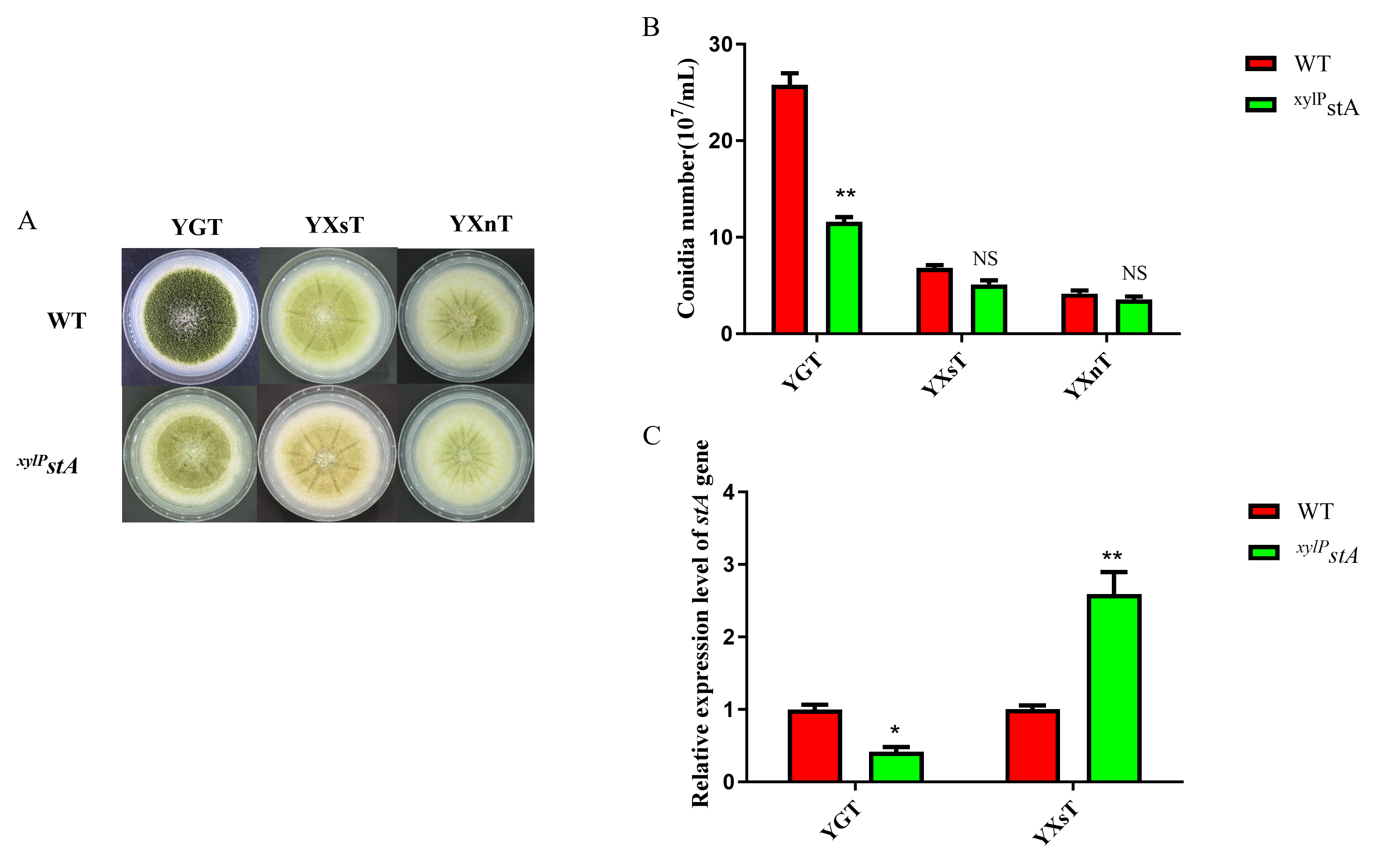

Supplement: Figure S3.tif [file KVIR_A_2532812_SM9891.tif]

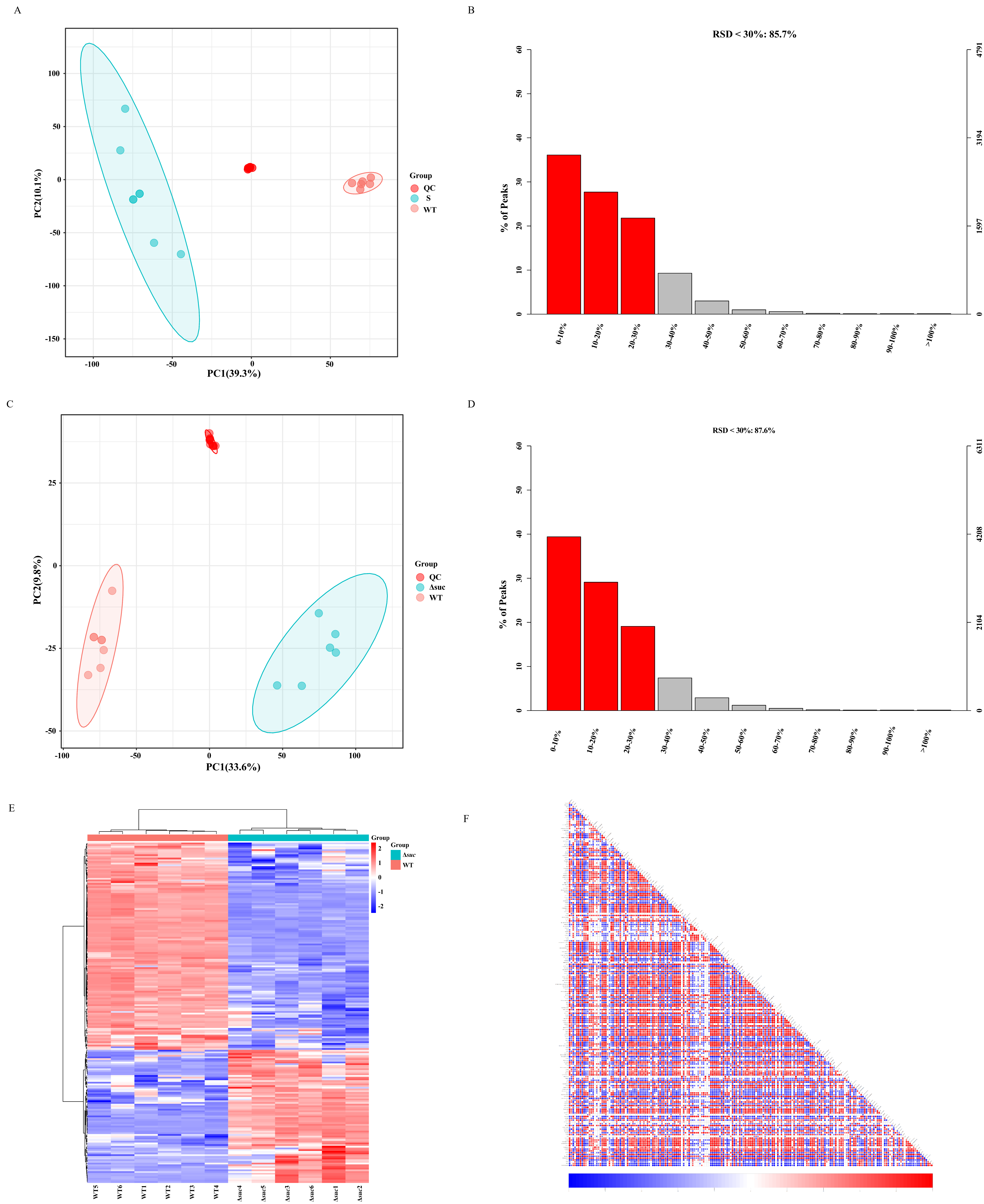

Supplement: Figure S6.tif [file KVIR_A_2532812_SM9890.tif]

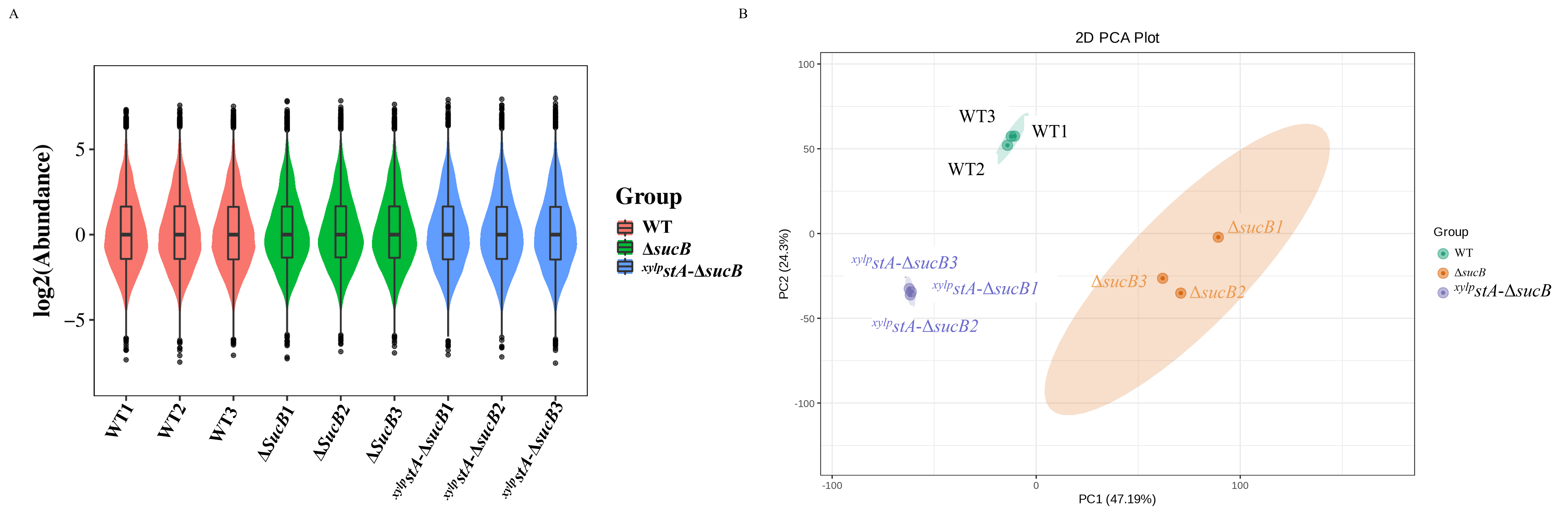

Supplement: Figure S4.tif [file KVIR_A_2532812_SM9889.tif]
